# Learning delays through gradients and structure: emergence of spatiotemporal patterns in spiking neural networks

**DOI:** 10.3389/fncom.2024.1460309

**Published:** 2024-12-20

**Authors:** Balázs Mészáros, James C. Knight, Thomas Nowotny

**Affiliations:** Sussex AI, School of Engineering and Informatics, University of Sussex, Brighton, United Kingdom

**Keywords:** spiking neural network, delay learning, dynamic pruning, receptive field, sparse connectivity

## Abstract

We present a Spiking Neural Network (SNN) model that incorporates learnable synaptic delays through two approaches: per-synapse delay learning via Dilated Convolutions with Learnable Spacings (DCLS) and a dynamic pruning strategy that also serves as a form of delay learning. In the latter approach, the network dynamically selects and prunes connections, optimizing the delays in sparse connectivity settings. We evaluate both approaches on the Raw Heidelberg Digits keyword spotting benchmark using Backpropagation Through Time with surrogate gradients. Our analysis of the spatio-temporal structure of synaptic interactions reveals that, after training, excitation and inhibition group together in space and time. Notably, the dynamic pruning approach, which employs DEEP R for connection removal and RigL for reconnection, not only preserves these spatio-temporal patterns but outperforms per-synapse delay learning in sparse networks. Our results demonstrate the potential of combining delay learning with dynamic pruning to develop efficient SNN models for temporal data processing. Moreover, the preservation of spatio-temporal dynamics throughout pruning and rewiring highlights the robustness of these features, providing a solid foundation for future neuromorphic computing applications.

## 1 Introduction

Spiking Neural Networks (SNNs) are the third generation of artificial neural networks (Maass, 1997), inspired by the functioning of biological neurons. Unlike traditional neural networks, which are *stateless* and process information through continuous activation values, neurons in SNNs are *stateful* and communicate via sparse binary spikes, mimicking the electrical impulses observed in biological neurons. This enables SNNs to efficiently process temporal information, making them well-suited to tasks involving sequential data processing. SNNs have shown significant potential as a computational paradigm for neuromorphic computing platforms (Furber et al., 2014; Davies et al., 2018; Merolla et al., 2014), enabling low-power and real-time processing. This makes them a compelling basis for next-generation intelligent systems.

Synaptic delays (between the emission of a spike and its arrival at the post-synaptic neuron) have been suggested as a means of improving the spatio-temporal information processing of SNNs (Izhikevich, 2006; Paugam-Moisy et al., 2008). These delays, which can vary across connections, allow neurons to perform coincidence detections across longer time intervals, enhancing SNN's ability to process temporal information. In biological systems, delays encompass axonal, synaptic, and dendritic components and are modified by processes like myelination to facilitate learning (Bengtsson et al., 2005) and coincidence detection (Seidl et al., 2010). Furthermore, neuromorphic hardware such as Intel's Loihi (Davies et al., 2018), SpiNNaker (Furber et al., 2014), and DenRAM (D'Agostino et al., 2024) incorporate programmable synaptic delays, enabling SNNs with delays to be efficiently deployed for real-time data processing. In the past, two kinds of delay learning methods have been used: delay selection and delay shift. Delay selection relies on implementing several synapses between each neuron with various delays, and picking the most optimal one (Bohte et al., 2002). Delay shift uses only a single synapse, and optimizes the corresponding delay. An early example of delay shift was the Delay Learning Remote Supervised Method (DL-ReSuMe) that showed improved performance compared to just training weights both in terms of accuracy and training speed (Taherkhani et al., 2015). Wang et al. (2019) further improved upon these results and Shrestha and Orchard (2018) extended the approach to enable synaptic delay learning in deep networks. In this paper, we use Dilated Convolutions with Learnable Spacings (DCLS) (Khalfaoui-Hassani et al., 2021) which, similarly to the approach proposed by Wang et al., convolves spike trains with delay kernels. However, DCLS can be used in deeper architectures and has been shown to be an effective delay learning method on neuromorphic benchmarks (Hammouamri et al., 2023).

Methods to decrease the number of parameters in a neural network were first proposed more than 35 years ago (LeCun et al., 1989; Hassibi et al., 1993) and, in recent years, overparametrisation in deep neural networks has become a widely acknowledged problem (Ba and Caruana, 2014). The lottery ticket hypothesis proposes that, within an overparameterised dense network, there exist multiple sparse sub-networks with varying performances and, among them, one sub-network stands out as the “winning ticket” that outperforms the others (Frankle and Carbin, 2018). Sparse neural networks significantly reduce memory usage and energy consumption and are *required* on many neuromorphic systems, which often have a maximum fan-in per neuron (Schemmel et al., 2010; Merolla et al., 2014) or limited memory available for connectivity (Furber et al., 2014; Davies et al., 2018). One means of obtaining a sparse network is to train a dense network and remove connections using a process called “pruning” (Han et al., 2015). However, this means that the size of models is still limited by the training cost of the original dense model. In contrast, biological systems dynamically rewire synaptic connections during learning, suggesting that dynamic pruning (also known as structure learning) and rewiring can enhance neural network performance and efficiency. The first algorithm that both disconnected and reconnected neurons during training was DEEP R (Bellec et al., 2018). The method drops synapses based on their weight changes during learning and replaces them with randomly chosen synapses to maintain a constant number of synapses. In parallel, another dynamic pruning method called sparse evolutionary training (SET) was introduced, relying purely on weight magnitudes to drop connections (Mocanu et al., 2018). Sparse Networks from Scratch (SNFS) used the momentum of each parameter as the criterion for reconnecting neurons (Dettmers and Zettlemoyer, 2019). RigL (Evci et al., 2021) took this one step further and uses gradient information for growing the network. These dynamic pruning methods aim to emulate biological efficiency, potentially offering superior sparsity and accuracy with fewer floating-point operations (FLOPs). According to calculations by Evci et al., at 90% sparsity, RigL requires 1/4 of FLOPs compared to the same size dense model, and DEEP R requires 1/10. With implementations exploiting sparsity, both algorithms can significantly speed up training and inference (Knight and Nowotny, 2023).

In this paper, we present our analysis of a spiking neural network trained in a supervised fashion on the Heidelberg Digits benchmark dataset (Cramer et al., 2022), preprocessed as described by Zenke and Vogels (2021). We analyze the learnt parameters of the fully connected network, then train networks with dynamic pruning and fixed sparse connectivity, and conduct the same analysis on these more efficient and biologically more plausible architectures.

## 2 Methods

We consider an SNN with Leaky-Integrate-and-Fire (LIF) neurons, solved with a linear Euler method,


(1) $$\begin{array}{l} u_{i}^{\left(l\right)}\left[t\right]=\left(1-\frac{\Delta t}{\tau }\right)u_{i}^{\left(l\right)}\left[t-1\right]\left(1-S_{i}^{\left(l\right)}\left[t-1\right]\right)+I_{i}^{\left(l\right)}\left[t\right]\cdot \Delta t\; \end{array}$$

(2) $$\begin{array}{l} S_{j}^{\left(l\right)}\left[t\right]=\Theta \left(u_{j}^{\left(l\right)}\left[t\right]-\vartheta \right), \end{array}$$

where, $u_{i}^{\left(l\right)}\left[t\right]$ is the membrane potential of the *i*-th neuron in layer *l* at time step *t*, τ = 10.05 is the membrane time constant and $I_{i}^{\left(l\right)}\left(t\right)$ is the input current. $S_{i}^{\left(l\right)}$ is the spike train emitted by neuron *i*, ϑ denotes the firing threshold and Θ the Heaviside function. For our numerical experiments, we used a timestep of Δ*t* = 1.

Because Θ is non-differentiable, we replace it with a arctan surrogate gradient during the backward pass of our training (Neftci et al., 2019). To implement the synaptic delay training, the input current $I_{i}^{\left(l\right)}\left(t\right)$ is calculated using DCLS (Hammouamri et al., 2023), i.e., convolving the spike train $S_{j}^{\left(l-1\right)}$ from layer *l*−1 with the 1D kernel


(3) $$\begin{array}{l} k_{ij}^{\left(l\right)}\left[n\right]=\frac{w_{ij}^{\left(l\right)}}{c}\exp\left(-\frac{1}{2}{\left(\frac{n-T_{d}+d_{ij}^{\left(l\right)}+1}{\sigma }\right)}^{2}\right),\;n=0,\ldots ,T_{d} \end{array}$$

where *T*_*d*_ = 25 is the maximum delay, $d_{ij}^{\left(l\right)}$ is the synaptic delay from neuron *j* to neuron *i* in layer *l*, *c* is a normalization term so that $\overset{T_{d}}{\underset{n=0}{\sum }}k_{ij}^{\left(l\right)}\left[n\right]=w_{ij}^{\left(l\right)}$ and σ = 12.5 is the standard deviation of the delay kernel, which is decreased during training. As the kernel $k_{ij}^{\left(l\right)}$ slides through the spike train $S_{j}^{\left(l-1\right)}$ at each timestep *t* the kernel will have access to spikes in the range of {*t*−*T*_*d*_, …, *t*}. The method essentially creates a temporal convolutional kernel, where the position of weights in the kernel corresponds to the synaptic delay. These weights can then be learned as normal, providing a framework in which weights and delays can be optimized together. For more details, we refer the reader to the original publication (Hammouamri et al., 2023).

For dynamic pruning, we combined two methods: DEEP R (Bellec et al., 2018) for dropping connections and RigL (Evci et al., 2021) for introducing them. In DEEP R the synaptic weights are defined as $w_{ij}^{l}=s_{ij}^{\left(l\right)}\text{max}\left(\theta_{ij}^{\left(l\right)},0\right)$, where $s_{ij}^{\left(l\right)}$ is a constant sign value, and $\theta_{ij}^{\left(l\right)}$ is the parameter trained with gradient descent. If $\theta_{ij}^{\left(l\right)}$ is not positive, the connection is considered dormant. We use *L*1 regularization to encourage pruning of unnecessary connections. Although the original DEEP R method adds noise to the gradients to induce stochasticity in ANNs, following Bellec et al. (2020), we omitted this in our experiments. DEEP R maintains a fixed number of synapses by randomly reactivating synapses throughout training.

RigL introduces synapses based on gradient analysis rather than randomly. In each iteration, the algorithm selects the *k* strongest negative gradients from the inactive connections:


(4) $$\begin{array}{l} \underset{\theta_{ij}^{\left(l\right)}\leq 0}{\mathrm{ArgTopK}}\left(-\frac{dL}{d\theta_{ij}^{\left(l\right)}},k\right). \end{array}$$

Since we make the assumption that the weight of active connections is positive, the original RigL method needs to be modified slightly. Instead of picking synapses to introduce based on the *absolute* gradient value, we simply pick based on the strongest negative gradient values, since a positive gradient implies that gradient descent wants to keep the connection inactive.

Most neural network models do not adhere to Dale's law, which states that neurons are either exclusively excitatory or inhibitory. Computationally, this means that the signs for the outgoing weights from each presynaptic neuron are the same. Since with DEEP R we have to generate the sign matrix before training, we can conveniently apply this constraint by generating a random sign vector *s*∈{−1, +1} and broadcasting it into a matrix. Unless stated otherwise, all of our results apply to networks that adhere to Dale's law.

To measure the spatial autocorrelation of the learned spatio-temporal patterns, we used the method of Moran's I (Moran, 1950):


(5) $$\begin{array}{l} I=\frac{N}{W}\frac{\sum_{i=1}^{N}\sum_{j=1}^{N}w_{ij}\left(x_{i}-\bar{x}\right)\left(x_{j}-\bar{x}\right)}{\sum_{i=1}^{N}{\left(x_{i}-\bar{x}\right)}^{2}} \end{array}$$

where *N* is the number of elements, *x* are the elements in the pattern, $\bar{x}$ is the mean of the elements, *w*_*ij*_ are the elements of the spatial weights with zero diagonals, and *W* is the sum of all *w*_*ij*_. We used the 8 neighborhood case (also known as the Queen's case) for the weights *w*_*ij*_ to capture a broad influence. Since the ordering in the spatial dimension is arbitrary (i.e. the ordering of the rows (neurons) can be changed), we took 2000 random row permutations of the matrix and determined the maximum Moran's I across them. With no spatial autocorrelation Moran's I is $\frac{-1}{N-1}$, meaning that it approaches 0 with increasing *N*. Moran's *I* values significantly below $\frac{-1}{N-1}$ indicates negative spatial autocorrelation whereas those significantly above $\frac{-1}{N-1}$ indicate positive spatial autocorrelation. We measure if the distributions of Moran's I values in trained and untrained networks are significantly different using the Mann-Whitney U test.

We extended the PyTorch implementation of DCLS developed by Hammouamri et al. (2023) for our experiments. Our architecture consisted of one hidden layer with 256 neurons, dropout layers with a probability of 0.4, batch normalization, and delays in all layers in the range of (0, …, 25) timesteps. We used a voltage sum readout and cross-entropy loss for training and our model was trained with the Adam optimizer, using a OneCycle scheduler for weights and a Cosine Annealing scheduler for delays.

## 3 Results

### 3.1 Spatio-temporal receptive fields of trained networks

**Figure 1:**
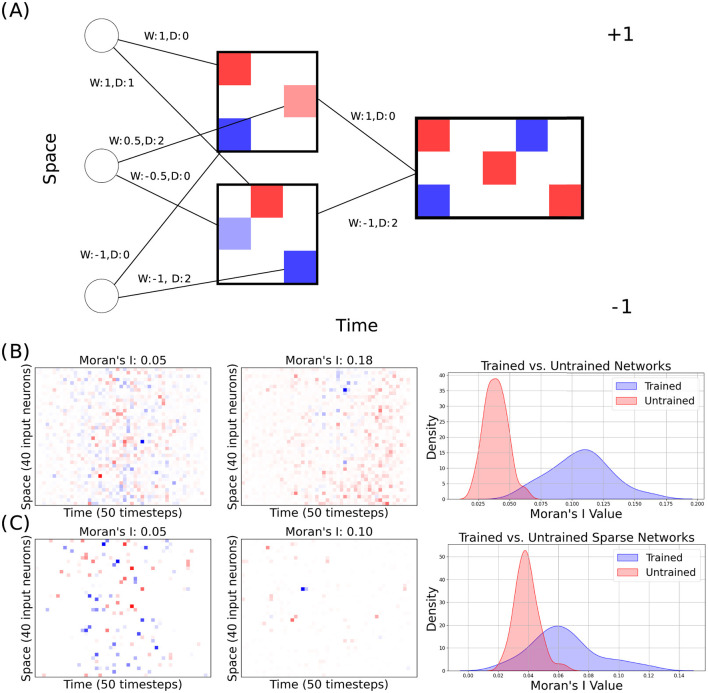
**(A)** Toy example of how the spatio-temporal receptive fields were generated. *W* denotes the synaptic strength, *D* denotes the synaptic delay. **(B)** The receptive field with the highest observed Moran's I value prior to training (left) and after training (middle) in a dense network. The distributions (right) show all observed Moran's I values. **(C)** The receptive field with the highest observed Moran's I value prior to training (left) and after training (middle) in a sparse network. The distributions (right) show all observed Moran's I values.

In our experiments, we trained our models with the best hyperparameters used by Hammouamri et al. (2023) for training on the Spiking Heidelberg Digits (SHD) dataset, but instead trained on the Raw Heidelberg Digits dataset (Cramer et al., 2022). We preprocessed the dataset similarly to Zenke and Vogels (2021), creating Mel spectrograms of shape 40 (input channels) by 80 (timesteps). We performed our experiments on this dataset because it carries complex enough temporal information to benefit from delay learning, but it can be solved with a relatively small network, which makes it better suited for our analysis. The only additional parameter we needed to tune was the *L*1 regularization strength. We first trained our model without any sparsity or Dale's law-derived constraints and achieved 97.2%. This is higher than the results reported by Zenke and Vogels (2021) (94 ± 2%) – the only other paper running benchmarks on RawHD. However, the goal of this paper is not benchmarking. We then analyzed the learnt spatio-temporal patterns by extracting what we refer to as “spatio-temporal receptive fields” using the process illustrated in Figure 1A. While, in most settings, receptive fields refer to *functional* attributes of the network (Linden et al., 2003; DeAngelis et al., 1995), we use a similar concept to analyse the *structural* attributes of a trained network in terms of the sign and delay of connections. For each hidden neuron, we created a panel with input neurons on the y-axis and delay along the x-axis. Then, for each input neuron, we place one point at the x coordinate corresponding to the learnt delay of its connection to the hidden neuron and colored either blue or red based on the weight's sign. We then summed these panels for each output layer neuron, weighting each by the learned hidden-to-output weight and aligning them on the x-axis according to the learned hidden-to-output delay. These figures allow us to see whether a certain feature for a given class is excited or inhibited and whether it takes effect immediately or with a delay. The results for the dense network before and after training are shown in Figure 1B. Visually, the figure illustrates that spatio-temporal patterns of excitation and inhibition formed after training.

### 3.2 Spatio-temporal autocorrelation of learned receptive fields

We assessed the spatio-temporal autocorrelation of each of the 20 output neuron's trained receptive fields by calculating the maximum Moran's I from 2,000 random row-wise permutations (as the spatial ordering of neurons in our architecture is arbitrary). We repeated our experiment 3 times and created distributions from the 3 × 20 receptive fields for trained and untrained networks (see Figure 1C). To assess whether there is a meaningful difference in spatio-temporal correlation within receptive fields between trained and untrained networks, we analyzed the distributions of Moran' I values for both network types using a Mann-Whitney U test. This non-parametric test was chosen because it does not assume a specific distribution shape for the data, making it suitable for comparing independent samples with potentially different variances and non-normality. For the dense networks, the Mann-Whitney U test yielded a test statistic of 3, 598.0 and a *p*-value of approximately 3.88 × 10^−21^. Given this very low *p*-value, we reject the null hypothesis (H0) that the distributions of Moran's I values in trained and untrained dense networks are identical. This result indicates a highly statistically significant difference between the two distributions with the trained dense networks exhibiting systematically different spatial correlations than the untrained networks.

### 3.3 Dynamic pruning

Next, we trained networks with dynamic pruning—utilizing DEEP R and RigL to enforce a fixed level of 87.5% sparsity and Dale's law by making all of each neuron's outgoing connections have the same sign. While, visually, the emergence of grouping is not as obvious as it was in the dense networks (Figure 1C), calculating Moran's I and performing a similar Mann-Whitney U test produced a test statistic of 3, 169.0 and a p-value of approximately 6.69 × 10^−13^. Although this p-value is higher than that observed for the dense network, it still provides strong evidence to reject the null hypothesis, indicating a statistically significant difference between the trained and untrained distributions for the sparse network.

These findings suggest that training introduces structural features within the network's receptive fields, contributing to increased spatial correlation that is absent in untrained networks. While the effect is more pronounced in dense networks, the presence of a significant difference in the sparse network, despite its high level of sparsity, highlights that spatial correlations remain an important outcome of the training process. This may imply that the network's learning captures and reinforces spatial dependencies critical to its task, as reflected in the elevated Moran's I values in trained networks.

### 3.4 Ablation study

**Figure 2:**
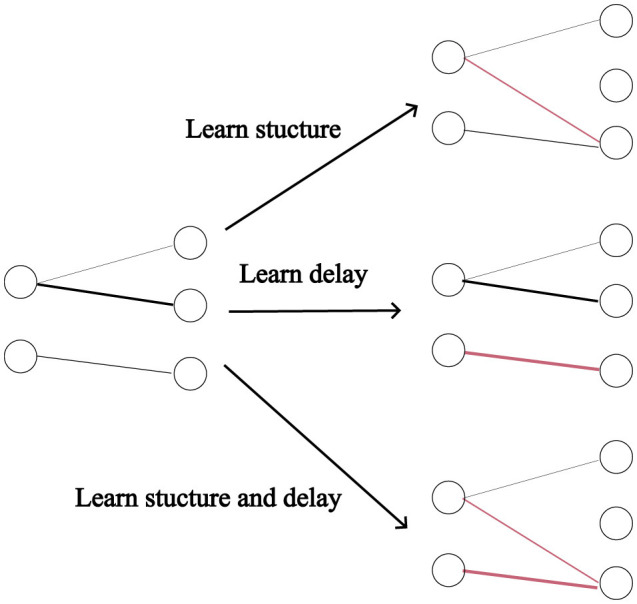
Types of learning. Delays are indicated by the thickness of the connections, and red is used to highlight the changes.

**Figure 3:**
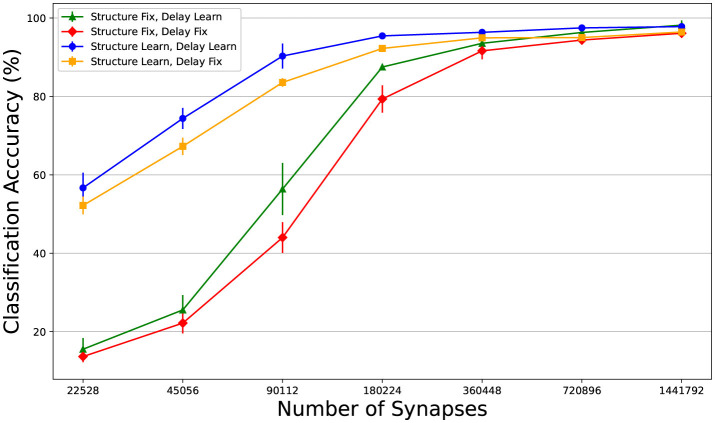
Comparing the effects of structure learning and delay learning. Learning the structure does not make a huge difference with lower sparsity, but as it increases the benefit becomes clear. This might not be that surprising, since these methods were built to train highly sparse models. Delay learning improves the results with a fixed connectivity matrix, as was also shown in (Hammouamri et al., 2023). The benefits of delay learning are not obvious when the structure is learnt.

In this section, we analyse the combined and separate usefulness of dynamic pruning (i.e. structure learning) and delay learning. We defined four models, one where the structure is learnt, one where the synaptic delays are learnt, one where both are learnt and one where neither are (the synaptic weights are still trained in all cases). See Figure 2 for illustration. For the simulations with fixed connectivity, we omitted Dale's law, since for the models with fixed connectivity, poor initialization could impose significant constraints on the network. Starting with a 50% sparsity, we progressively halved the number of connections in a sequence of experiments. The effectiveness of DEEP R and RigL seems highly dependent on initialization, so we ran three repeats of each configuration and reported the average classification accuracy. Figure 3 demonstrates that dynamic pruning is highly effective, especially as sparsity increases. While learning delays is beneficial when the structure is fixed, its benefits are less obvious when the structure is learnt. Overall, dynamic pruning seems vital for maintaining high performance in sparse networks and we would argue that, when structure is learned, delay learning might not be necessary.

## 4 Discussion

SNNs have a strong potential for spatio-temporal processing and synaptic delays only enhance this. Our study demonstrates a new approach for the visual analysis of networks with delay learning, revealing that functional spatio-temporal patterns emerge in both dense and sparse networks. These patterns appear more in networks where the framework allows for the optimization of temporal parameters through gradients or structural learning. We compared the Moran's I distributions of models trained with and without delay learning and dynamic pruning. The mean Moran's I value of the model with fixed delays and structure was much lower (0.027 compared to 0.064) and the distributions were significantly different (Mann-Whitney U test statistic of 166.5 and p-value of approximately 9.97 × 10^−18^). At the same time the classification performance is less (see Figure 3, red line), suggesting that emerging spatio-temporal structure and classification success correlate.

While delay learning seems useful with sparse connectivity, we found that learning the structure is more important. In fact, when the structure was learnt, delay learning appeared to bring little benefit. This may be because gradient descent struggles with simultaneously learning both parameters; if a newly connected synapse has a non-optimized delay, it might be immediately deactivated again. However, our experiments with using RigL for removing connections (which does not happen every epoch like DEEP R) did not show significant benefits, challenging this theory. From another perspective, structure learning with fixed delays *is* a form of delay learning since, if a synapse has an ineffective delay, the network can adapt by introducing a more effective synapse with a new random delay. This method closely resembles “delay select” SNNs (Bohte et al., 2002). However, for this method to work in the fully connected setting, several connections are required between each pre and postsynaptic neuron, with the number increasing as the delay distribution widens. Ergo, from the point of view of efficiency, we have an argument for delay learning methods such as the one based on DCLS, especially in the fully connected setting. However, we argue that a good test for the usefulness of a given delay learning method is whether, in a sparse network, it performs better than simply replacing synapses with new ones that have a fixed random delay. In our experiments, we relied on gradient information to reconnect weights, but this only yielded minor improvements over random reconnection (standard DEEP R). This implies that, in our simulations, randomly sampling a delay value was just as effective as adjusting delays using gradient descent. While both in our experiments and the ones run by Hammouamri et al. (2023), delay learning does improve network performance, perhaps the precision that delay gradient information provides is not necessary and slows down learning performance.

In conclusion, our results show that sparse network architectures can be efficient for machine learning tasks. Our findings pave the way for future research into the optimization of SNNs for various applications, particularly those at the edge that have strong memory and computational constraints.

## Data Availability

The raw data supporting the conclusions of this article will be made available by the authors, without undue reservation. Source code is available at: https://github.com/mbalazs98/spatiotemporal.

## References

[B1] Ba J. Caruana R. (2014). Do deep nets really need to be deep? *Advances in Neural Information Processing Systems* (San Diegeo, CA: Neural Information Processings Systems Foundation), 27.

[B2] Bellec G. Kappel D. Maass W. Legenstein R. (2018). Deep rewiring: training very sparse deep networks. arXiv [preprint] arXiv:1711.05136. 10.48550/arXiv.1711.05136

[B3] Bellec G. Scherr F. Subramoney A. Hajek E. Salaj D. Legenstein R.. (2020). A solution to the learning dilemma for recurrent networks of spiking neurons. Nat. Commun. 11:3625. 10.1038/s41467-020-17236-y 32681001 PMC7367848

[B4] Bengtsson S. L. Nagy Z. Skare S. Forsman L. Forssberg H. Ullén F. (2005). Extensive piano practicing has regionally specific effects on white matter development. Nat. Neurosci. 8, 1148–1150. 10.1038/nn1516 16116456

[B5] Bohte S. M. Kok J. N. La Poutre H. (2002). Error-backpropagation in temporally encoded networks of spiking neurons. Neurocomputing 48, 17–37. 10.1016/S0925-2312(01)00658-0

[B6] Cramer B. Stradmann Y. Schemmel J. Zenke F. (2022). The heidelberg spiking data sets for the systematic evaluation of spiking neural networks. IEEE Trans. Neural Netw. Learn. Syst. 33, 2744–2757. 10.1109/TNNLS.2020.3044364 33378266

[B7] D'Agostino S. Moro F. Torchet T. Demiraúg Y. Grenouillet L. Castellani N.. (2024). DenRAM: neuromorphic dendritic architecture with RRAM for efficient temporal processing with delays. Nat. Commun. 15:3446. 10.1038/s41467-024-47764-w 38658524 PMC11043378

[B8] Davies M. Srinivasa N. Lin T.-H. Chinya G. Cao Y. Choday S. H.. (2018). Loihi: A neuromorphic manycore processor with on-chip learning. IEEE Micro 38, 82–99. 10.1109/MM.2018.112130359

[B9] DeAngelis G. C. Ohzawa I. Freeman R. D. (1995). Receptive-field dynamics in the central visual pathways. Trends Neurosci. 18, 451–458. 10.1016/0166-2236(95)94496-R 8545912

[B10] Dettmers T. Zettlemoyer L. (2019). Sparse networks from scratch: faster training without losing performance. arXiv [preprint] arXiv:1907.04840. 10.48550/arXiv.1907.04840

[B11] Evci U. Gale T. Menick J. Castro P. S. Elsen E. (2021). Rigging the lottery: making all tickets winners. arXiv [preprint] arXiv:1911.11134. 10.48550/arXiv.1911.11134

[B12] Frankle J. Carbin M. (2018). The lottery ticket hypothesis: Finding sparse, trainable neural networks. arXiv [preprint] arXiv:1803.03635. 10.48550/arXiv.1803.03635

[B13] Furber S. B. Galluppi F. Temple S. Plana L. A. (2014). The SpiNNaker project. Proc. IEEE 102, 652–665. 10.1109/JPROC.2014.2304638

[B14] Hammouamri I. Khalfaoui-Hassani I. Masquelier T. (2023). Learning delays in spiking neural networks using dilated convolutions with learnable spacings. arXiv preprint arXiv:2306.17670. 10.48550/arXiv.2306.17670

[B15] Han S. Pool J. Tran J. Dally W. (2015). “Learning both weights and connections for efficient neural network,” in Advances in Neural Information Processing Systems (San Diegeo, CA: Neural Information Processings Systems Foundation), 28.

[B16] Hassibi B. Stork D. G. Wolff G. J. (1993). “Optimal brain surgeon and general network pruning,” in IEEE International Conference On Neural Networks (San Francisco, CA: IEEE), 293–299.

[B17] Izhikevich E. M. (2006). Polychronization: computation with spikes. Neural Comput. 18, 245–282. 10.1162/089976606775093882 16378515

[B18] Khalfaoui-Hassani I. Pellegrini T. Masquelier T. (2021). Dilated convolution with learnable spacings. arXiv [preprint] arXiv:2112.03740. 10.48550/arXiv.2112.03740

[B19] Knight J. C. Nowotny T. (2023). “Easy and efficient spike-based machine learning with mlGeNN,” in Proceedings of the 2023 Annual Neuro-Inspired Computational Elements Conference (New York, NY: ACM), 115–120.

[B20] LeCun Y. Denker J. Solla S. (1989). “Optimal brain damage,” in Advances in Neural Information Processing Systems (San Diegeo, CA: Neural Information Processings Systems Foundation), 2.

[B21] Linden J. F. Liu R. C. Sahani M. Schreiner C. E. Merzenich M. M. (2003). Spectrotemporal structure of receptive fields in areas ai and aaf of mouse auditory cortex. J. Neurophysiol. 90, 2660–2675. 10.1152/jn.00751.2002 12815016

[B22] Maass W. (1997). Networks of spiking neurons: the third generation of neural network models. Neural Netw. 10, 1659–1671. 10.1016/S0893-6080(97)00011-7

[B23] Merolla P. A. Arthur J. V. Alvarez-Icaza R. Cassidy A. S. Sawada J. Akopyan F.. (2014). A million spiking-neuron integrated circuit with a scalable communication network and interface. Science 345, 668–673. 10.1126/science.1254642 25104385

[B24] Mocanu D. C. Mocanu E. Stone P. Nguyen P. H. Gibescu M. Liotta A. (2018). Scalable training of artificial neural networks with adaptive sparse connectivity inspired by network science. Nat. Commun. 9:2383. 10.1038/s41467-018-04316-3 29921910 PMC6008460

[B25] Moran P. A. (1950). Notes on continuous stochastic phenomena. Biometrika 37, 17–23. 10.1093/biomet/37.1-2.17 15420245

[B26] Neftci E. O. Mostafa H. Zenke F. (2019). Surrogate gradient learning in spiking neural networks. arXiv [preprint] arXiv:1901.09948. 10.48550/arXiv.1901.09948

[B27] Paugam-Moisy H. Martinez R. Bengio S. (2008). Delay learning and polychronization for reservoir computing. Neurocomputing 71, 1143–1158. 10.1016/j.neucom.2007.12.027

[B28] Schemmel J. Brüderle D. Grübl A. Hock M. Meier K. Millner S. (2010). “A wafer-scale neuromorphic hardware system for large-scale neural modeling,” in 2010 IEEE International Symposium on Circuits and Systems (ISCAS) (Paris: IEEE), 1947–1950.

[B29] Seidl A. H. Rubel E. W. Harris D. M. (2010). Mechanisms for adjusting interaural time differences to achieve binaural coincidence detection. J. Neurosci. 30, 70–80. 10.1523/JNEUROSCI.3464-09.2010 20053889 PMC2822993

[B30] Shrestha S. B. Orchard G. (2018). “Slayer: Spike layer error reassignment in time,” in Advances in Neural Information Processing Systems (San Diegeo, CA: Neural Information Processings Systems Foundation), 31.

[B31] Taherkhani A. Belatreche A. Li Y. Maguire L. P. (2015). Dl-resume: a delay learning-based remote supervised method for spiking neurons. IEEE Trans. Neural Netw. Learn. Syst. 26, 3137–3149. 10.1109/TNNLS.2015.2404938 25794401

[B32] Wang X. Lin X. Dang X. (2019). A delay learning algorithm based on spike train kernels for spiking neurons. Front. Neurosci. 13:252. 10.3389/fnins.2019.00252 30971877 PMC6445871

[B33] ZenkeF.VogelsT. P. (2021). The remarkable robustness of surrogate gradient learning for instilling complex function in spiking neural networks. Neural Comput. 33, 899–925. 10.1162/neco_a_0136733513328

